# The Effects of the Incorporation of Nano-Hydroxyapatite on Physico-Chemical Properties of Calcium-Enriched Mixed Cement 

**DOI:** 10.30476/dentjods.2024.101837.2319

**Published:** 2025-06-01

**Authors:** Mohammadreza Nabavizadeh, Yasser Samadi, Safoora Sahebi, Fateme Eskandari

**Affiliations:** 1 Oral and Dental Disease Research Center, Dept. of Endodontics, School of Dentistry, Shiraz University of Medical Sciences, Shiraz, Fars, Iran.; 2 Dept. of Endodontics, Oral Health Center, Health Research Institute, School of Dentistry, Babol University of Medical Sciences, Babol, Iran.; 3 Dept. of Endodontics, School of Dentistry, Shiraz University of Medical Sciences, Shiraz, Fars, Iran.; 4 Dentist, School of Dentistry, Shiraz University of Medical Sciences, Shiraz, Iran.

**Keywords:** Solubility, Compressive strength, pH, Calcium silicate, Nanoparticles

## Abstract

**Background:**

Calcium-enriched mixed (CEM) cement, though beneficial in endodontic applications, requires improvements in its physico-chemical properties to enhance clinical outcomes.

**Purpose:**

This study was conceptualized to investigate the impact of incorporating nano-hydroxyapatite (nHAP) on the physico-chemical properties of CEM cement.

**Materials and Method:**

In this experimental study, nHAP powder at 5 and 10 wt% ratio was thoroughly mixed with CEM cement powder. Then they were mixed with a ratio of 1 g of powder to 0.33g of liquid and placed in special molds for each test. CEM cement without nHAP was used as a control sample. Samples were assessed for setting time, compressive strength, solubility, and pH.

**Results:**

According to our results, the addition of 5% nHAP significantly increased the initial setting time (S1) and compressive strength after 24 hours, while the addition of 5% and 10% nHAP significantly enhanced the pH of the CEM cement.

**Conclusion:**

Incorporation of 5% nHAP to CEM cement, although delayed S1 of this cement, but increased the pH level of CEM cement, which in turn could potentially improve the antimicrobial properties of CEM cement. Furthermore, the addition of 5% nHAP to CEM cement notably improved the compressive strength in the short term, which can be beneficial in withstanding the chewing forces after applying the cement in the oral environment. It is recommended to select an appropriate concentration of nHAP to optimize the properties of CEM cement based on the findings of this study.

## Introduction

Calcium silicate based cements (CSCs) belong to a group of the hydrophilic self-hardening cements [ 1 ]. CSCs powder is mainly composed of di and tricalcium silicate. Upon the mixing of the powder, calcium hydroxide and hydrated calcium silicate are predominantly produced, leading to the formation of a viscous colloidal gel (hydrated calcium silicate gel) that ultimately solidifies into a rigid structure [ 2 - 3 ]. This type of cement is frequently utilized in various endodontic procedures, like pulp regeneration, root-end filling, repair of the perforation, apexogenesis, pulp capping, pulpotomy, and apexification [ 4 ]. 

In 2006, Asgari *et al*. [ 5 ] introduced a novel endodontic cement known as CEM cement for the purpose of root-end filling. This biomaterial exhibits favorable physical characteristics like setting time, flow, and layer thickness . CEM cement can generate hydroxyapatite (HAP) in saline solution, potentially promoting stem cell differentiation and induction of the formation of hard tissue . Additionally, it can set in aqueous environments more quickly than mineral trioxide aggregate (MTA) and demonstrates similar sealing ability . The clinical indications of CEM cement closely resemble those of MTA, showing comparable results when used for pulp capping or perforation repair [ 12 - 13 ]. Moreover, it possesses an antibacterial effect similar to calcium hydroxide and exhibits superior antibacterial properties compared to MTA or Portland cement (PC) [ 14 ]. The physical properties of CEM cement are according to ISO 2001 standards and are acceptable to experts and this endodontic cement seems to be a suitable root-end filling due to appropriate consistency, efficiency, compatibility and setting time [ 7 ].

CEM is chemically different from MTAs and PCs; phosphorus is a main constituent of CEM, while it is present in small amounts in MTAs and PCs and the surface composition of CEM cement is more identical to dentin than calcium silicate based materials (CSMs) [ 15 ]. Considering that hydroxyapatite (HAP) is a primary component of dentin, the similarity between CEM cement and dentin may enhance the bonding and cementation process [ 8 ]. This novel cement liberates calcium and phosphorus ions, resulting in an abundant supply of OH-, Ca2+ and PO4 ions. These components are involved in the production of HAP [ 16 ]. Biocompatibility is important feature of endodontic materials [ 17 ]. The biocompatibility of CEM is related to its potential to liberate calcium ions during setting, and subsequently calcium to phosphorus binding to form HAP crystals . 

HAP is a calcium phosphate compound represented by the molecular formula Ca10 (PO4) OH2. Pure HAP typically consists of 39.68% calcium and 18% phosphorus by weight, leading to a Ca/P molar ratio of 1.67 [ 18 ]. Crystalline HAP is present in bones and teeth, with HAP crystals comprising approximately 65 to 70% of bone weight as a bioactive ceramic [ 19 ]. Due to its advantageous physico-chemical and mechanical properties, nHAP has become widely utilized in dental applications [ 17 ]. The primary drawback of pure HAP lies in its porous structure and inadequate mechanical properties, making it relatively weak and brittle for load-bearing applications [ 20 ]. Consequently, HAP is often used in combination with a composite or polymer matrix to enhance its practical use [ 21 - 22 ]. 

Crystalline HAP nanoparticles are more medically important than crystalline HAP microparticles due to their greater similarity to bone HAP [ 23 ]. The nanoscale varies from 1 to 200 nanometers, in which a certain activity of the particles is obtained. As the particle size diminishes and the reactivity level increases, the hydration of the material also increases, resulting in better physical and chemical properties [ 24 ].

Nanoparticles have been utilized to enhance both the mechanical and biological properties of numerous dental materials [ 25 - 27 ]. Over the course of nearly two decades, nHAP has been incorporated into various dental materials, such as glass ionomer, MTA, and calcium hydroxide, due to its biocompatible, bioactive, and antimicrobial properties. Overall, the inclusion of nHAP in dental materials in numerous studies has yielded positive outcomes in the properties under investigation. Additionally, alkaline salts, chlorhexidine, and sodium hypochlorite have been integrated into CEM cement to enhance its physical and chemical attributes [ 28 - 34 ].

So far, no study has been performed to evaluate the effects of adding nHAP to CEM cement on its physico-chemical properties. This study aims to investigate the impact of incorporating nHAP on the physico-chemical properties of CEM cement. 

## Materials and Method

In the current study, CEM cement (Yektazdandan; Bionique Dent, Tehran, Iran) and nHAP (EC Number: 215-145-7, CAS: 1306-06-5, chemical formula: Ca₅(PO₄)₃ OH, Nanosadra; Mashhad, Iran) were utilized. nHAP powder was incorporated into CEM cement powder in ratios of 5 and 10 weight percent and then thoroughly mixed by an amalgamator (Farazmehr; Esfahan, Iran) at 4500 rpm. According to previous studies, in order to match the samples, one gram of powder was mixed with 0.33 g of CEM cement liquid [ 35 ]. The dough was then pressed into the molds with a force of 100 grams for each experiment, and any excess material was removed using a wet cotton. CEM cement without nHAP was used as a control sample. The samples were distributed into 3 groups including CEM cement, CEM +5% nHAP, and CEM +10% nHAP.

### Evaluation of setting time

To assess the setting time, stainless steel rings measuring 10mm in inner diameter and 1 mm in thickness (n= 6) were prepared for filling with various materials. Subsequently, the samples were placed in a fully-sealed plastic vial and kept in an incubator at 100% humidity and 37°C for 150±1 seconds. The evaluation of setting time was conducted in accordance with ISO-6876 [ 36 ] and ASTM [ 37 ] standards. Evaluations were conducted at the following intervals: every 3 minutes for the first 30 minutes, every 5 minutes for the subsequent 90 minutes, and every 15 minutes thereafter, starting from two hours. For the determination of initial setting time (S1), a Gilmore needle weighing 100±0.5 g and with a tip diameter of 2±0.1mm was employed to test the material's surface. This process was repeated at three separate points until the tip of the device no longer affected the material's surface. After determining S1, the measurements were continued until the final setting of the CEM cement. To determine the final setting time (S2) of these steps, a Gilmore needle with a mass of 456±0.5 g and a tip diameter of 1±0.1 mm was used and this was repeated until the tip of the device could no longer affect the surface of the material at three independent points.

### Evaluation of compressive strength

Samples (n= 6) were prepared according to BSI-6039 standard [ 38 ] for compressive strength test. The materials were placed in cylinders with a diameter of 6 mm and a height of 12 mm and the samples were placed at 37°C and 100% humidity for 3 hours. The specimens were subsequently taken out of the cylinder and then subjected to further incubation for 24 hours and 21 days. Compressive strength test was conducted using Emic DL2000 device (Zwick/Roell Z020 Zwick, CombH & Co, Germany) with a force of 5000 N and a speed of 0.5 mm per minute. The maximum stress in MPa was recorded using the maximum compressive force (p) and cylinder diameter (d), based on the following formula (MPa=N/mm^2^). $\mathrm{MPa}=\frac{\mathrm{4p}}{\pi d^{2}}$

### Solubility evaluation

Solubility assessment was done according to ISO-6876 standard [ 28 ]. Samples (n= 10) were made in diameter of 7mm and 1mm thick and a 5cm nylon thread was inserted into the samples when the samples were placed in the mold. The samples were then incubated in two groups for 2 and 7 days at 100% humidity and 37°C. After 2 and 7 days following the setting time, the samples were taken out of the mold, and after removing the additives and loose particles, the samples were placed in the silica desiccator for one hour. The sample disk with the attached nylon thread was then measured three times with an accurate scale and the average was recorded as the initial mass. Each sample was then stored in a 10 ml tank containing deionized water at 37°C for 15 hours. After this period, the samples were taken out of the tank and washed with distilled water and deionized water. The samples were placed in a silica desiccator for 24 hours. Thereafter, the final mass was determined by conducting three measurements of the samples, with the average value recorded for analysis. The solubility of the material corresponds to the amount of mass lost; it is calculated as a percentage of the initial mass based on the following formula: 

$\mathrm{Solubility (\%):}=\frac{W_{1}-W_{2}}{W_{1}}\times \mathrm{100}$

W_1_: Dry weight of cement before being placed in water

W_2_: Dry weight of cement after being in water for 15 hours

### Evaluation of pH

The pH evaluation was carried out according to Faria-Junior *et al*. study [ 39 ]. Samples (n= 10) were made in dimensions of 7mm in diameter and 1mm thick and incubated at 100% humidity and 37°C for 2 and 7 days until the setting time was completed. The samples were then immersed in a plastic flask containing 10 ml of deionized water in pH= 6.5. The flask was sealed and re-incubated at 37°C. The pH was measured by pH meter (HANNA pH Tester, HI98100 Cheker *plus; Woonsocket; Romania) at intervals of 5 and 15 hours following immersion of the samples. 

### Data analysis

The data was inputted into SPSS software version 22, and a normality test was conducted for all experiments to assess normal distribution. Subsequently, for comparison between different groups, parametric tests including the ANOVA statistical test and Tukey's multiple comparison test were performed on the data, using a significance level of 0.05.

### Study samples and materials

The study design was approved by ethical committee of Shiraz University of Medical Sciences (code IR.SUMS. DENTAL. REC.1399.057). 

## Results

### Setting time

As Table 1 shows, S1 had the shortest time in CEM cement without nHAP (*p*< 0.05). While in S2, the shortest time belonged to CEM +5% nHAP group, although it was not statistically significant (*p*= 0.369). 

**Table 1:** Mean and standard deviation of setting time in minutes

Setting time (min)	S1	S2
Cement	Mean±(SD*)	Mean±(SD)
CEM	65.8 (10.6)^a^	190 (8.9)^d^
CEM +5% nHAP*	84.1 (5.8)^b^	186 (10.3)^d^
CEM +10% nHAP	105 (16.4)^c^	195 (10.4)^d^

The same letters in each row and column indicate statistical similarity, *p*< 0.05 *SD: standard deviation nHAP: nano hydroxyapatite

### Compressive strength

The results (Table 2) showed that in the period of 24 hours after setting, adding 5% nHAP to CEM cement significantly increased the compressive strength. There was a significant difference with CEM group and CEM +5% nHAP group (*p*< 0.05). However, during the same period, addition of 10% nHAP to CEM cement, slightly reduced the compressive strength, which was not significantly different from the CEM group (*p*= 0.43). In the period of 21 days after setting, the addition of 5% nHAP to CEM cement slightly increased the compressive strength, but the addition of 10% nHAP to CEM cement slightly decreased the compressive strength. Overall, the addition of 10% nHAP to CEM cement did not result in statistically significant changes (*p*= 0.452). 

**Table 2:** Mean and standard deviation of compressive strength in MPa

Time elapsed since setting	24 hours	21 days
Cement	Mean±(SD*)	Mean±(SD)
CEM	1.52 (0.87)^a^	36.03 (11.85)^c^
CEM +5% nHAP*	2.69(0.44)^b^	37.39(7.93)^c^
CEM +10% nHAP	1.07 (0.36)^a^	30.98 (6.44)^c^

The same letters in each row and column indicate statistical similarity, *p*< 0.05 *SD: standard deviation nHAP: nano hydroxyapatite

### pH 

The results indicated that the incorporation of 5 and 10 % nHAP to CEM cement increased the pH significantly in both experimental periods (2 and 7 days after setting) and at intervals of 5 and 15 hours after immersion in de-ionized water (*p*< 0.05). This increase in pH was proportional to the increase in nHAP. In addition, the results showed that all groups had a higher pH level in the first hours after mixing compared to the longer period (15 hours). The highest pH was observed in the CEM +10% nHAP group in the experimental period 2 days after setting and 5 hours after immersion in deionized water (Table 3). 

**Table 3:** Mean and standard deviation of pH

Immersion time	2 days after setting		7 days after setting	
Cement	5 hours Mean± (SD*)	15 hours Mean± (SD)	5 hours Mean± (SD)	15 hour Mean± (SD)
CEM	10.88(0.155)^a^	10.55(0.186)^d^	10.51(0.091)^g^	10.24(0.096)^j^
CEM+5% nHAP*	11.25(0.108)^b^	11(0.141)^e^	10.78(0.173)^h^	10.64(0.249)^h^
CEM+10% nHAP	11.48(0.105)^c^	11.34(0.130)^f^	11.03(0.996)^i^	10.94(0.135)^k^

The same letters in each row and column indicate statistical similarity, *p*<0.05 *SD: standard deviation nHAP: nano hydroxyapatite

### Solubility

The highest solubility was obtained by adding 10% nH-AP to CEM cement in 2 days after setting, which was decreased over time (7 days after setting) in all groups, and was not statistically significant (*p*= 0.350) (Table 4). 

**Table 4:** Mean and standard deviation of solubility in percentage of initial mass

Time elapsed since setting	2 days	7 days
Cement	Mean±(SD*)	Mean±(SD)
CEM	5.50(1.93)^a^	4.29(1.31)^a^
CEM +5% nHAP*	6.30(1.80)^a^	5.38(1.94)^a^
CEM +10% nHAP	7.05(2.86)^a^	5.57(2.74)^a^

The same letters in each row and column indicate statistical similarity, *p*< 0.05 *SD: standard deviation nHAP: nano hydroxyapatite

## Discussion

In the current study, the mean S1 in the CEM group was 65.8 minutes and in the CEM group+5% nHAP was 84.1 minutes and in the CEM group+10% nHAP was 105 minutes. Abbaszadegan *et al*. [ 28 ] reported that the mean setting time in CEM cement was 58.3 minutes and Asgari *et al*. [ 40 ] indicated a setting time of less than one hour (approximately 50 minutes) which was almost in accordance with the findings of the present study. Abbaszadegan *et al*. [ 28 ] examined the impact of introducing calcium chloride to CEM. Their findings demonstrated a notable decrease in the mean setting time as a result of the addition of calcium chloride [ 28 ]. However, in the present study, the addition of nHAP caused a significant increase in S1. In fact, increasing setting time after adding nHAP to different cements has been reported in previous studies. Lee *et al*.'s study [ 41 ] showed that the setting time of glass ionomer cement increased significantly after adding nHAP. They also reported that nHAP had a greater effect on increasing setting time compared to its microparticles [ 41 ]. This raise in setting time may be due to increased surface area and reactivity of nHAP particles, which increases the hydration of the material and increases the ratio of liquid to powder [ 24 ]. In a similar study on calcium hydroxide cement, Yasaei *et al*. [ 48 ] indicated that the setting time of this cement in the presence of nHAP increased significantly, which was directly related to the amount (percentage) of nanoparticles added, which is consistent with our findings. In other words, less change was observed in the CEM+5% nHAP group than in the CEM+10% nHAP.

In our study, S1 recorded the shortest time for CEM cement without nHAP (*p*< 0.05). In contrast, the shortest time in S2 was observed in the CEM+5% nHAP group, although this difference was not statistically significant. Guerreiro-Tanomaru *et al*. [ 42 ] demonstrated that the PC+ZrO2+HAn10% group had the shortest final setting time, whereas the MTA group exhibited the longest [ 42 ]. It seems that the prolongation of the setting time is due to the interruption of the setting reaction, after incorporating greater values of nHAP. The manufacturer has not reported the exact ratio of liquid to powder and has announced the best ratio when a creamy consistency is obtained. This causes errors and inconsistencies between the studies. However, the reason for the difference between these two investigations with the current study could be due to the difference in the liquid to powder ratio, the diameter of the inductor and the amount of force applied during recording setting time. 

During filling the root canal, alkaline environment is a key factor which helps to improve pulp tissue, improve mineralization process, stop resorption process and increase antibacterial properties [ 43 ]. In the current study, at 2 and 7 days after setting the cement, incorporation of nHAP to CEM cement significantly increased the pH level, which was higher than CEM at both immersion times of 5 and 15 hours. These findings indicate that the addition of nHAP increases the alkalinity of CEM cement and this increase in pH is related to the amount of nHAP added. The highest pH in the CEM+ 10% nHAP group in 5 hours after immersion in deionized water (pH= 11.48). Elevated pH levels have antibacterial properties against common endodontic pathogens. A pH range of 10.5-11.0 can impede the growth of E. faecalis, while at a pH of 11.5, no growth of the bacteria occurs [ 31 ]. This important event was achieved by adding nHAP to CEM cement in the present study.

Increased alkaline activity by adding calcium chloride to CEM cement was also observed in the study of Abbaszadegan *et al*. [ 28 ]. The overall amplitude of pH increase in the present study was consistent with the results of previous studies for CEM and CSCs in which the maximum pH increase occurred in the early hours after immersion and then remained constant or decreased over time [ 28 , 34 , 44 ]. 

 A number of studies on other cements show opposite results. Guerreiro-Tanomaru *et al*. [ 42 ] showed that the incorporation of nHAP to Portland cement and zirconium oxide (PC+ZrO2) did not cause a significant increase in pH. Moreover, Antonijevic *et al*. [ 45 ] reported that nHAP had no significant effect on the pH of calcium silicate cement. Also, Zamanian *et al*. [ 46 ] indicated that the incorporation of 5% nHAP to calcium hydroxide cement slightly reduced the pH [ 46 ]. These inconsistencies may be related to the chemical composition of cements, the concentration of nHAP, or the experimental conditions.

The compressive strength is a key physical characteristic of hydrophilic cements, and it is associated with their hydration process [ 47 ]. In the present study, two-time intervals (24 hours and 21 days) were used to measure compressive strength. Shorter time was chosen due to the importance of initial compressive strength in clinical applications (exposure to occlusal forces after exposure to the patient's oral environment). Similar periods have been used in previous studies for this purpose [ 42 , 46 - 47 ]. A 21-day duration was chosen to assess the impact of nHAP addition over an extended period. The results from both the longer and shorter periods were compared with those of the control group. Long-term strength plays a vital role in resisting occlusal forces and the forces generated during the placement of restorative materials [ 49 ]. 

Eskandarinezhad *et al*. [ 50 ] showed that MTA cement in combination with nHAP showed higher compressive strength in 4 days after mixing than pure MTA cement, although this difference was not statistically remarkable but on 21 days after testing, the compressive strength of both groups was very close, indicating that nHAP has no significant effect on the compressive strength of MTA cement in the long time. Over a 21-day period, these findings were consistent with the results of our study because the addition of nHAP reduced the compressive strength of CEM cement but was not statistically significant. Zamanian *et al*. [ 46 ] reported that adding 5% nHAP notably improved the compressive strength of cement, but adding more than this amount had the opposite effect. Therefore, they recommended that the optimal size of the added nanoparticles should be considered [ 46 ]; these findings were consistent with the results of our study. The compressive strength of CEM cement was optimally enhanced by incorporating 5% nHAP in the short term, which could be due to the addition of nHAP and lower porosity in new cement, high density of interfaces, increased hydration over time, higher specific surface area, and higher crystallinity . In addition, in this study it was observed that by adding nHAP up to 10%, the compressive strength is imperceptibly reduced, it can be suggested that adding more than 5% nHAP to the cement matrix may disrupt slowly the setting reaction and homogeneous dispersion of particles [ 42 , 46 , 48 ]. 

Solubility refers to the number of released particles that are washed away by the solution or solvent, resulting in a reduction in cement weight [ 54 ]. The durability and clinical success and of cements in the oral cavity rely on characteristics like structural integrity and dimensional stability that are commensurate with the water uptake reaction and solubility [ 55 ]. Factors influencing solubility include the chemical composition of the solvent, immersion duration, temperature, amount of unreacted substrate, as well as the size and chemical composition of leachable materials [ 56 ]. 

In this study, the solubility rate increased at both 2 and 7 days after cement setting by adding nHAP to CEM cement with both 5 and 10% ratios that was the highest in CEM+10% nHAP group, although this difference between different groups and times was not statistically significant. The elevated solubility can be attributed to the incomplete setting reaction of the cement caused by the chemical composition of the additives [ 57 ].

Shojaei *et al*. [ 58 ] reported that increasing liquid to powder ratio, significantly increases the solubility of CEM cement. Guerreiro-Tanomaru *et al*. [ 42 ] also reported that the addition of nHAP to Portland cement significantly increased the solubility of this cement. In the study of Abbaszadegan *et al*. [ 28 ], the addition of calcium chloride to CEM cement significantly reduced the solubility of this cement. Shojaee *et al*. [ 59 ] indicated that there was no significant difference between the solubility of MTA and CEM at 7 and 28 days after exposure to the synthesized tissue fluid. They showed that the solubility of CEM at times 7 and 28 was 0.5% and 3%, respectively. Not declaring about the exact ratio of liquid to powder and suitable test environment by the manufacturer, the addition of nanoparticles that increase hydration, and the number of nanoparticles added, may result in interfering with the setting reactions of cement and disrupt the homogeneous dispersion of particles. They can be a reason for the increase in solubility in these studies and in higher percentages of nHAP.

This study has some limitations that should be considered when interpreting the results. Firstly, the evaluation was conducted only over a short-term period, and thus, the long-term performance of CEM cement with nHAP incorporation remains uncertain. Additionally, in vivo testing was not performed, which limits our understanding of how these findings translate to clinical settings. Future research should focus on long-term assessments and clinical trials to better ascertain the durability and effectiveness of nHAP-enhanced CEM cement in practical applications. 

## Conclusion

The addition of 5% nHAP to CEM cement delayed the initial setting time (S1) but significantly increased the pH level and compressive strength in the short term. This enhancement may improve the antimicrobial properties of CEM cement and its ability to withstand chewing forces in the oral environment.
